# Tesaglitazar ameliorates non-alcoholic fatty liver disease and atherosclerosis development in diabetic low-density lipoprotein receptor-deficient mice

**DOI:** 10.3892/etm.2012.713

**Published:** 2012-09-17

**Authors:** BU-CHUN ZHANG, WEI-MING LI, XIAN-KAI LI, MENG-YUN ZHU, WEN-LIANG CHE, YA-WEI XU

**Affiliations:** Department of Cardiology, Shanghai Tenth People’s Hospital, Tongji University School of Medicine, Shanghai 200072, P.R. China

**Keywords:** atherosclerosis, type 2 diabetes, mice, non-alcoholic fatty liver disease, peroxisome proliferator-activated receptor, inflammation

## Abstract

Previous research has demonstrated that the dual PPARα/γ agonist tesaglitazar reduces atherosclerosis in a mouse model of hyperlipidemia by reducing both lipid content and inflammation in the aorta. However, much of the underlying mechanism of tesaglitazar in non-alcoholic fatty liver disease (NAFLD) remains less clear. The aim of the present study was to determine whether tesaglitazar attenuates NAFLD and atherosclerosis development in diabetic low-density lipoprotein receptor-deficient (LDLr^−/−^) mice. Female LDLr^−/−^ mice (3 weeks old) were induced by a high-fat diet (HFD) combined with low-dose streptozotocin (STZ) injection to develop an animal model of type 2 diabetes (T2DM). The mice were randomly divided into two groups: diabetic group (untreated diabetic mice, n=15) and tesaglitazar therapeutic group (n=15, 20 μg/kg/day oral treatment for 6 weeks). Fifteen LDLr^−/−^ mice were fed with an HFD as the control group. Tesaglitazar decreased serum glucose and lipid levels compared with the diabetic mice. Tesaglitazar significantly reduced atherosclerotic lesions, lipid accumulation in the liver, macrophage infiltration, and decreased total hepatic cholesterol and triglyceride content compared to the diabetic mice. In addition, tesaglitazar reduced inflammatory markers at both the serum and mRNA levels. Our data suggest that tesaglitazar may be effective in preventing NAFLD and atherosclerosis in a pre-existing diabetic condition by regulating glucose and lipid metabolism, and the inflammatory response.

## Introduction

The prevalence of metabolic syndrome associated with visceral obesity is increasing worldwide, resulting in a high risk of developing complications, such as diabetes and cardiovascular diseases. These pathologies are pathophysiologically associated with atherosclerosis and non-alcoholic fatty liver disease (NAFLD), which exhibit common features such as lipid accumulation and inflammation (1,2). These features develop silently over several years, and are the most common causes of cardiovascular and chronic liver diseases.

Atherosclerosis and NAFLD progression are both accompanied by inflammation development which involves several factors, including various chemokines (monocyte chemoattractant protein 1; MCP-1), cytokines (tumor necrosis factor-α; TNF-α), interleukin-6 (IL-6) and C-reactive protein (CRP) (3–6). NAFLD and atherosclerosis are now recognized as two aspects of a shared disease, involving the local presence of activated macrophages (7). Macrophages are able to accumulate large amounts of lipids, transform into foam cells and drive atherogenesis (8,9). The same process of accumulation of lipid-loaded macrophages was observed in NAFLD, where hyperlipidemic mice demonstrated bloated foamy Kupffer cells (KCs) upon high-fat feeding (10). Consequently, drugs targeting lipid homeostasis and inflammation may have great potential to improve NAFLD and reduce atherosclerosis development.

Peroxisome proliferator-activated receptors (PPARs) are nuclear receptors that control the expression of genes involved in both carbohydrate and lipid metabolism, and could be valuable as additional drug targets (11). PPARα regulates the expression of genes encoding proteins that are involved in lipid metabolism, fatty acid oxidation and glucose homeostasis, thereby improving markers for atherosclerosis and insulin resistance. In addition, PPARα exerts anti-inflammatory effects both in the vascular wall and the liver (12). PPARγ agonists improve insulin sensitivity and induce glycaemic control in diabetic animals as well as in patients with type 2 diabetes (13). Thus, compounds that stimulate both PPARα and PPARγ (dual PPARα/γ agonists) should additively improve both lipid and glucose abnormalities in animal models and human subjects with insulin resistance (IR) and/or type 2 diabetes (T2DM). However, the effects of dual PPARα/γ agonists on NAFLD have not been thoroughly studied.

The aim of the present study was to determine whether dual PPARα/γ agonist tesaglitazar attenuates NAFLD and atherosclerosis development in diabetic low-density lipoprotein receptor-deficient (LDLr^−/−^) mice. Female LDLr^−/−^ mice were induced by a high-fat diet (HFD) combined with a low-dose STZ injection to develop an animal model of T2DM, and to induce NAFLD and atherosclerotic lesions.

## Materials and methods

### Generation of the diabetic model and treatment

All animal procedures were performed in compliance with ‘The Guide for the Care of Use of Laboratory Animals’ published by the National Institute of Health (NIH Publication No. 85-23, revised 1996) and approved by the Animal Care Committee of Shanghai Tenth People’s Hospital, Tongji University School of Medicine. The mice were housed five per cage and allowed access to the appropriate diet and autoclaved water.

Three-week-old female LDLr^−/−^ mice with a C57BL/6 genetic background were created by homologous recombination (Jackson Laboratory, Bar Harbor, ME, USA) and were fed an HFD (20% fat, 20% sugar and 1.25% cholesterol). After 6 weeks, the LDLr^−/−^ mice underwent an intraperitoneal glucose tolerance test (IPGTT). Mice exhibiting IR were injected once with low-dose streptozotocin (STZ, 75–80 mg/kg i.p.) to induce partial insulin deficiency. Two weeks after the STZ injection, most HFD/STZ-treated mice displayed hyperglycemia, IR and glucose intolerance, as previously reported (14). At age 11 weeks, animals with similar degrees of hyperglycemia and body weight were randomly divided into untreated (DM, n=15) and tesaglitazar-treated (DM+tesaglitazar, n=15) groups. The mice fed an HFD were used as a nondiabetic control group (n=15). For tesaglitazar treatment, the mice received a daily oral dose of tesaglitazar (20 μg/kg/day). These drugs were dissolved in water and administered by oral gavage. The dose of drug was calculated based on previous studies (15,16). After 6 weeks of treatment, the mice were sacrificed under isoflurane anesthesia, and tissues were removed and frozen immediately in liquid nitrogen or fixed with paraffin. Blood samples were collected from the mice for measurement of serum parameters.

### Biochemical studies

Blood samples were collected from mice at the beginning of the experiment, before pharmacological triggering and at the end of the experiment. Fasting serum glucose, total cholesterol (TC), triglyceride (TG) and high-density lipoprotein-cholesterol (HDL-C) were measured using commercial kits (Sigma, St. Louis, MO, USA). Serum MCP-1, TNF-α, IL-6 and CRP were measured using commercial enzyme-linked immunosorbent assay (ELISA) kits purchased from R&D Systems, Inc. (Minneapolis, MN, USA).

### Analysis of atherosclerotic lesions

After sacrifice by cervical dislocation, hearts were fixed with 4% phosphate-buffered paraformaldehyde (PAF), and 10-μm aortic sinus sections were cut followed by quantitative analysis of lipid deposition by Oil Red O staining. Aortic sinuses were also stained with rat monoclonal anti-mouse macrophage Mac3 (1:100, BD Biosciences, Heidelberg, Germany), followed by detection with biotinylated secondary antibody and streptavidin-horseradish peroxidase. Images were captured using a JVC 3-charge-coupled device video camera (Sony, Tokyo, Japan) and analyzed using the computer-assisted Quips Image analysis system (Leica Microsystems GmbH, Germany).

### Histological analysis of the liver

At sacrifice, livers were perfused with phosphate buffered saline (PBS) solution via the portal vein. After removal of the liver, a section of approximately 4 mm^2^ was fixed in PAF and embedded in paraffin. Paraffin-embedded sections (5 μm) were stained with hematoxylin and eosin (H&E) to evaluate steatosis and inflammation. Frozen-liver sections (7 μm) were stained with rat monoclonal anti-mouse macrophage F4/80 (1:500, Serotec, Oxford, UK), followed by detection with biotinylated secondary antibody and streptavidin-horseradish peroxidase, to evaluate macrophage infiltration. Analysis of lipid deposition was performed by Oil Red O staining of 7-μm frozen-liver sections, with H&E staining for visualization of the nuclei.

### Hepatic lipid analysis

Frozen liver tissue (50 mg) was homogenized in SET buffer (1 ml; sucrose 250 mM, EDTA 2 mM and Tris 10 mM), followed by two freeze-thaw cycles, three cyles through a 27-gauge syringe needle and a final freeze-thaw cycle. TG and TC were measured as described above.

### Real-time quantitative reverse transcription-polymerase chain reaction

Total RNA was isolated from mouse livers using the acid guanidinium thiocyanate/phenol/chloroform method. RNA was reverse-transcribed using Moloney murine leukemia virus reverse transcriptase and random hexamer primers (Invitrogen, Carlsbad, CA, USA). mRNA levels were quantified by real-time quantitative PCR on a MX-3000 apparatus (Agilent) using the Brilliant SYBR Green QPCR master mix (Qiagen, Shanghai, China). The following primers were constructed: TNF-α, 5′CGGAGTCCGGGCAGGT3′ (forward) and 5′GCTGGGTAGAGAATGGATGAACA3′ (reverse); MCP-1, 5′CAGCCAGATGCAGTTAACGC3′ (forward) and 5′GCCTACTCATTGGGATCATCTTG3′ (reverse); IL-6, 5′TGAGGAGACTTGCCTG3′ (forward) and 5′ACAACAACAATCTGAGGTGCC3′ (reverse). Individual gene expression was normalized to glyceraldehyde 3-phosphate dehydrogenase (GAPDH) expression. All quantifications were performed in triplicate samples for three separate experiments.

### Statistical analysis

Values are presented as means ± SD unless indicated otherwise. All data analysis was performed with the use of GraphPad PRISM statistical software 5.0 (San Diego, CA, USA). The statistical significance of differences between groups was determined by one-way ANOVA followed by the Dunnett’s multiple comparison test, or Student’s t-test when comparisons were made between 2 groups. P<0.05 was considered to indicate a statistically significant result.

## Results

### Effect of tesaglitazar on metabolic parameter change

As shown in Table I, at the beginning of the experiment, there was no significant differences in serum TC, TG and glucose levels, and body weight among the three groups of mice. Prior to pharmacological triggering, all diabetic mice had higher serum TC and TG and glucose levels than levels in the control group (P<0.001). The mean body weight was significantly higher for diabetic mice than HFD-fed mice at the age of 17 weeks (P<0.01). After 6 weeks of oral tesaglitazar treatment, serum TC, TG and glucose levels decreased in the diabetic mice (P<0.001). In addition, tesaglitazar treatment led to a significant increase in serum HDL levels in the diabetic mice (P<0.001). However, tesaglitazar treatment caused an increase in body weight (P<0.01).

### Effect of tesaglitazar on serum inflammatory markers

All diabetic mice had higher serum MCP-1, TNF-α, IL-6 and CRP levels than the control group (all P<0.001). As shown in Table II, after 6 weeks of oral tesaglitazar treatment, serum inflammatory marker levels were significantly reduced in diabetic mice (P<0.001, P<0.01, P<0.05).

### Effect of tesaglitazar on atherosclerotic lesions

**Figure 1:**
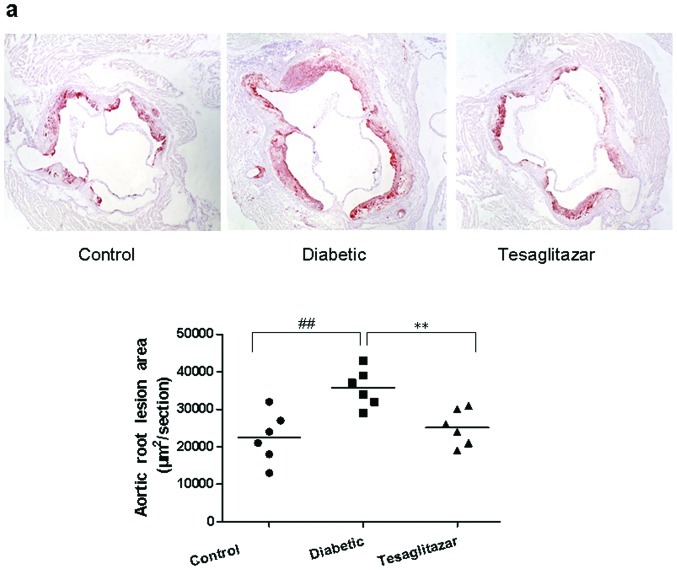

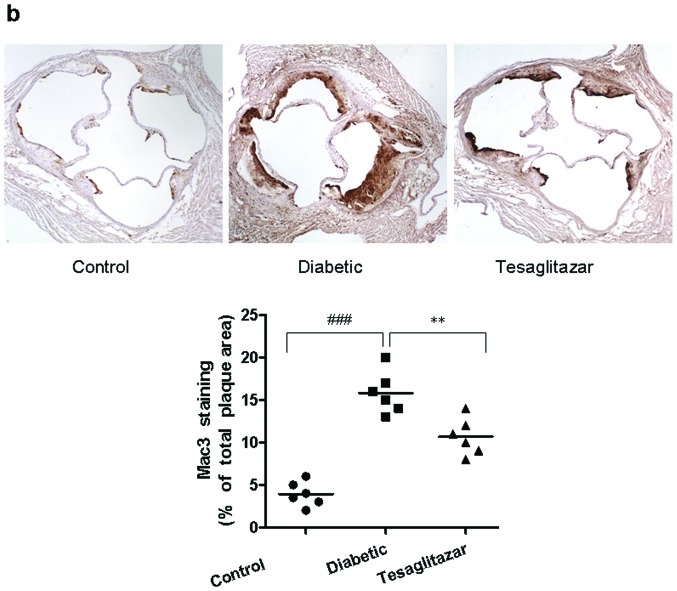
Effect of tesaglitazar on atherosclerotic lesion size and macrophage content in diabetic low-density lipoprotein receptor-deficient mice of the control, diabetic and tesaglitazar treatment diabetic groups. (a) Atherosclerotic lesion area was quantified after Oil Red O staining. (b) The macrophage content of atherosclerotic plaques. Original magnification, x40. Data represent the means ± SE (n=9). ^###^P<0.001, ^##^P<0.01, diabetic group vs. control group; ^**^P<0.01, diabetic group vs. tesaglitazar group.

Oil-red O staining of atherosclerotic plaques was red and widely observed in the diabetic mice. Compared with the size of plaque in the control mice, plaques were increased in the diabetic mice (P<0.01) but significantly diminished in the tesaglitazar-treated diabetic mice (P<0.01; Fig. 1a). Macrophage expression in the atherosclerotic plaques was identified by immunohistochemical staining of Mac3. The Mac3-positive area was larger in the diabetic mice group compared with the tesaglitazar-treated diabetic mice (P<0.01) and control mice groups (P<0.001; Fig. 1b).

### Effect of tesaglitazar on NAFLD

**Figure 2:**
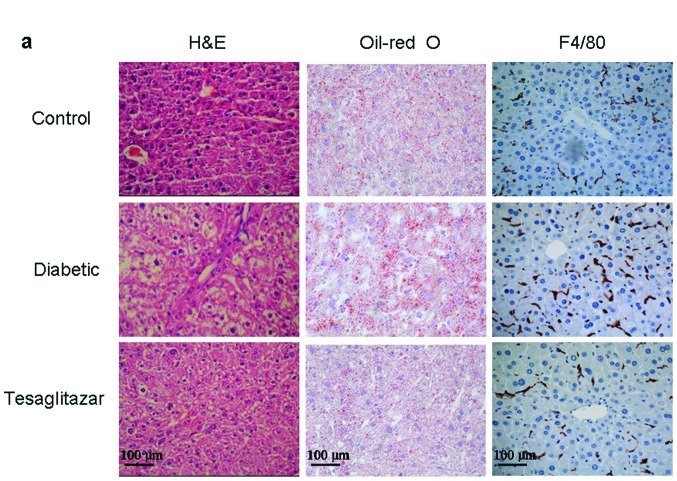

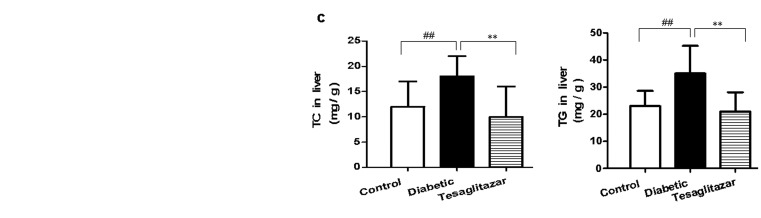
Effect of tesaglitazar on non-alcoholic fatty liver disease in diabetic low-density lipoprotein receptor-deficient mice of the control, diabetic and tesaglitazar treatment groups. (a) Hepatic steatosis and degree of inflammation were visualized by hematoxylin and eosin (H&E), Oil-red O staining and F4/80 immunohistochemistry. Original magnification, x200. (b) The number of F4/80-positive cells was quantified. (c) Hepatic lipid profile. TC, total cholesterol; TG, triglyceride. Data represent the means ± SE (n=6). ^###^P<0.001, ^##^P<0.01, diabetic group vs. control group; ^**^P<0.01, diabetic group vs. tesaglitazar group.

To investigate the inhibitory effect of tesaglitazar on NAFLD, we analyzed the histology of liver tissue using H&E staining, Oil-red O staining and F4/80 immunohistochemistry. As shown in Fig. 2a and b, marked microvesicular steatosis accompanied by a partial mild inflammation was observed in diabetic mice. However, the degree of hepatic fat accumulation was substantially alleviated by tesaglitazar treatment and the number of infiltrating macrophages (F4/80-positive cells) was significantly reduced in the tesaglitazar-treated diabetic mice group (P<0.01). In addition, tesaglitazar treatment reduced total hepatic cholesterol and triglyceride content (both P<0.01; Fig. 2c).

### Effect of tesaglitazar on inflammatory genes in the liver

**Figure 3:**
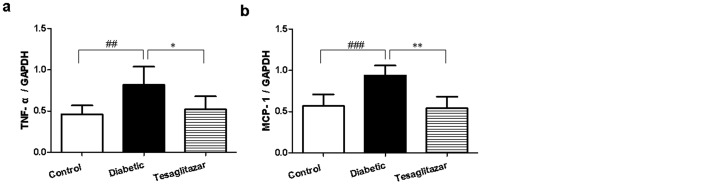
Tesaglitazar regulates the expression of inflammation-related genes in the liver, as demonstrated by quantitative real-time PCR analysis. (a) TNF-α, tumor necrosis factor-α. (b) MCP-1, monocyte chemoattractant protein 1. (c) IL-6, interleukin-6. Data represent the means ± SE (n=6). ^###^P<0.001, ^##^P<0.01 diabetic group vs. control group; ^**^P<0.01, ^*^P<0.05 diabetic group vs. tesaglitazar group.

In comparison with control mice, TNF-α, MCP-1 and IL-6 mRNA expression was significantly increased in the liver of diabetic mice (P<0.01, P<0.001, P<0.001), but this was reduced following 6-week treatment with tesaglitazar (P<0.01, P<0.05; Fig. 3).

## Discussion

Ubiquitously expressed, dual PPARα/γ agonists have been implicated in lipid metabolism and energy homeostasis. Previous studies have established that tesaglitazar treatment reduces atherosclerosis in a mouse model of hyperlipidemia by reducing both lipid content and inflammation in the aorta (15,16), yet the details regarding the underlying mechanism of tesaglitazar in NAFLD remain less clear.

To address this issue, we examined the effect of tesaglitazar on HFD-induced hepatic steatosis in diabetic LDLr^−/−^ mice. According to our data, lipid accumulation was significantly reduced by treatment with tesaglitazar when compared with that of the diabetic mice. These data indicate the potential of tesaglitazar as a therapeutic agent for HFD-induced fatty liver.

In the present study, the effects of tesaglitazar on serum lipid concentrations were similar to those in previous studies (15,16), namely TC and TG reduction, and HDL-C increase. Tesaglitazar improved serum glucose disorders observed in this animal model.

NAFLD and atherosclerosis are characterized by lipid accumulation and chronic inflammation (17,18). Numerous studies have reported NAFLD as an important cardiovascular risk factor (19,20), Kleemann *et al* demonstrated that after 10 weeks of a high cholesterol diet (1% cholesterol), the lesion area in female APOE*3-Leiden (E3L) mice was correlated with plasma levels of serum amyloid A protein (SAA), suggesting that the hepatic inflammatory response is involved in the formation of early atherosclerotic lesions (21). Due to the prominent role of the liver in the uptake of lipids, the hepatic inflammatory response in hyperlipidemic models precedes plaque formation. Increasing evidence suggests a shared metabolic and inflammatory factors associated with macrophage activation in atherosclerosis and NAFLD (22,23) and thereby supports the proposal that NAFLD and atherosclerosis are actually two aspects of the same disease.

As inflammation plays a pivotal role in both atherosclerosis and NAFLD, an important pharmacological objective in both disorders is to target inflammatory activation directly. The role of PPARs as anti-inflammatory mediators has been well-established. In the present study, we demonstrated that dual PPARα/γ agonist tesaglitazar inhibited macrophage infiltration both in the aortic root and in the liver. Our data also demonstrated that treatment with tesaglitazar decreased serum concentrations of MCP-1, TNF-α, IL-6 and CRP. Tesaglitazar also reduced the mRNA levels of TNF-α, MCP-1 and IL-6 in the liver of diabetic mice. These inflammatory markers were previously shown to be involved in the inflammatory cascade, for example, IL-6 is a multifunctional cytokine that plays a critical role in the acute-phase inflammatory response and its expression is known to be stimulated upon TNF-α-mediated activation of NF-κB and promotes the expression of intercellular adhesion molecule 1 (ICAM-1) and CRP synthesis. IL-6 has also been shown to stimulate macrophages to secrete MCP-1 (24).

Due to the complexity of the mechanisms that cause tissue lipid accumulation, we were unable to identify a single major factor or mechanism responsible for the observed inhibitory effect of tesaglitazar on hepatic lipid accumulation in our experiment. There are numerous possible mechanisms that may explain the observed effect of tesaglitazar in the present study and such mechanisms include food intake, energy expenditure, or fat accumulation and expression of fatty acid oxidation enzymes in other tissues. We could not test all the hypotheses and that was the major limitation of the present study. However, our findings suggest that dual PPARα/γ agonist tesaglitazar inhibits the development of NAFLD and atherosclerosis in a diabetic mouse model by regulating glucose and lipid metabolism, and the inflammatory response. Additional studies are required to clarify the mechanisms by which tesaglitazar induces these effects *in vitro*.

## Figures and Tables

**Table I:** Average weights, cholesterol, triglyceride and glucose levels (n=15, mean ± SD).

Group	Body weight (g)	Glu (mg/dl)	TC (mg/dl)	TG (mg/dl)	HDL (mg/dl)
Baseline (at age 3 weeks)					
Control	22.3±1.4	118.7±17	487.4±82	175.6±34	
Diabetic	22.1±1.1	114.1±15	483.1±90	173.2±27	
Before intervention (at age 11 weeks)					
Control	30.5±1.6	124.1±15	1025.6±74	395.6±10	
Diabetic	30.9±1.2	273.7±84^b^	1351.3±126^b^	816.0±88^b^	
Tesaglitazar	30.7±1.4	272.5±79	1354.7±123	814.2±85	
After intervention (at age 17 weeks)					
Control	39.6±1.2	130.5±17	1323.0±65	624.0±30	117.2±8
Diabetic	41.2±1.7^a^	300.5±80^b^	2114.3±118^b^	1113.2±58^b^	92.3±6^b^
Tesaglitazar	43.5±2.1^c^	152.6±23^d^	1478.7±123^d^	715.6±33^d^	105.7±3^d^

Glu, glucose; TC, total cholesterol; TG, triglyceride; HDL, high-density lipoprotein.

a P<0.01 and

b P<0.001, diabetic group vs. control group;

c P<0.01 and

d P<0.001, diabetic group vs. tesaglitazar group.

**Table II:** Serum inflammatory markers as measured by enzyme-linked immunosorbent assay in mice of the three groups after 6-week intervention (n=15, mean ± SD).

Groups	MCP-1 (pg/ml)	TNF-α (ng/ml)	IL-6 (pg/ml)	CRP (μg/ml)
Control	87.2±12.3	2.1±0.6	129.6±14.5	2.1±0.3
Diabetic	122.4±23.1^b^	3.4±0.2^b^	205.2±23.7^b^	3.4±0.2^b^
Tesaglitazar	102.7±19.6^c^	2.7±0.4^e^	174.3±26.1^d^	2.0±0.4^e^

MCP-1, monocyte chemoattractant protein 1; TNF-α, tumor necrosis factor-α; IL-6, interleukin 6; CRP, C-reactive protein.

a P<0.01 and

b P<0.001, diabetic group vs. control group;

c P<0.05,

d P<0.01 and

e P<0.001, diabetic group vs. tesaglitazar group.
